# N-Acetyl Cysteine Modulates the Inflammatory and Oxidative Stress Responses of Rescued Growth-Arrested Dental Pulp Microtissues Exposed to TEGDMA in ECM

**DOI:** 10.3390/ijms21197318

**Published:** 2020-10-03

**Authors:** Gili Kaufman, Drago Skrtic

**Affiliations:** ADA Science & Research Institute, LLC, Department of Innovation & Technology Research, Hagerstown, MD 21742, USA; skrticd@ada.org

**Keywords:** N-Acetyl Cysteine, rescue, dental pulp, 3D/ECM cell cultures, spatial growth, inflammation, stress response

## Abstract

Dental pulp is exposed to resin monomers leaching from capping materials. Toxic doses of the monomer, triethyleneglycol dimethacrylate (TEGDMA), impact cell growth, enhance inflammatory and oxidative stress responses, and lead to tissue necrosis. A therapeutic agent is required to rescue growth-arrested tissues by continuing their development and modulating the exacerbated responses. The functionality of N-Acetyl Cysteine (NAC) as a treatment was assessed by employing a 3D dental pulp microtissue platform. Immortalized and primary microtissues developed and matured in the extracellular matrix (ECM). TEGDMA was introduced at various concentrations. NAC was administered simultaneously with TEGDMA, before or after monomer addition during the development and after the maturation stages of the microtissue. Spatial growth was validated by confocal microscopy and image processing. Levels of inflammatory (COX2, NLRP3, IL-8) and oxidative stress (GSH, Nrf2) markers were quantified by immunoassays. NAC treatments, in parallel with TEGDMA challenge or post-challenge, resumed the growth of the underdeveloped microtissues and protected mature microtissues from deterioration. Growth recovery correlated with the alleviation of both responses by decreasing significantly the intracellular and extracellular levels of the markers. Our 3D/ECM-based dental pulp platform is an efficient tool for drug rescue screening. NAC supports compromised microtissues development, and immunomodulates and maintains the oxidative balance.

## 1. Introduction

Tissue repair is a well-orchestrated and complex process. Inflammatory cells multitask at the wound site by producing cytokines, chemokines, metabolites, and growth factors. The dysregulation and excess of inflammation are associated with impaired wound healing [1]. Non-healing wounds do not progress through the normal phases of wound repair and remain in a chronic inflammatory state [2]. Local stimuli can extend the inflammatory phase of the healing process and trigger a cascade of tissue responses that, in turn, may cause organ failure and/or tissue death [3]. The chronic wound is a highly pro-oxidant microenvironment and various reactive oxidant species (ROS) released into the wound environment have a prominent role in non-healing wound pathogenesis [4]. ROS cause direct damage to the cell membrane and structural proteins of the extracellular matrix which eventually leads to the expression of proinflammatory mediators [5,6]. As an outcome, the disturbed oxidant/antioxidant balance in the chronic wound is considered a major factor that amplifies the inflammatory state of the wound [2,4]. 

Triethylenglycol demethylate (TEGDMA), a frequently employed dental comonomer [7], is often released from the incompletely polymerized/eroded dental restoratives used for dental pulp capping [8,9,10,11]. TEGDMA can diffuse through dentinal tubules into the pulp chamber at high concentrations (millimolar range [12,13]). Direct as well as indirect pulp therapy, can cause cytotoxicity, cell cycle arrest, impaired healing, inflammation, and necrosis of pulp tissue [14,15]. The hydrophilicity/lipophilicity of leachable comonomer enables its penetration into biological environments and cells [8,16]. TEGDMA reportedly suppresses cellular vitality, changes cellular structures, provokes ROSs production, induces glutathione depletion, and induces pulpal inflammation [17,18]. At concentrations 1.5 to 3.0 mmol/L, TEGDMA affects pulp cell growth by increasing the number of cells arrested in G1 and G2 cycles [18,19] and interferes with the development of their progenitor dental papilla microtissues in a dose-dependent manner. The latter is due to the prevention of cell interaction and the creation of the spherical and inter-spherical interactions in the extracellular matrix microenvironment [20].

Oxidative stress is a condition when pro-oxidant/antioxidant imbalance occurs due to abnormally high levels of free radicals and/or a decline in antioxidant defense mechanisms [21,22,23]. Oxidative stress is strongly associated with the increased ROS and free radical formation leading to cell and tissue damage [24,25]. Oxidation is increased in both acute and chronic wounds [26] and related to the inflammatory reaction [6,27] during which damage of the tissue occurs due to pro-oxidant/antioxidant imbalance [28]. Therefore, excessive oxidative stress should be treated with antioxidants to achieve an anti-inflammatory response. Dental monomers were found to induce oxidative stress in human dental pulp cells and the depletion of the antioxidant glutathione (GSH) [29,30,31,32].

N-Acetyl Cysteine (NAC) is a derivative of the amino acid L-cysteine and glutathione (GSH) precursor [33,34]. NAC has been Food and Drug Administration (FDA)-approved as a therapeutic drug for a wide range of disorders [35,36]. NAC exhibits anti-inflammatory activity by moderating the synthesis of inflammatory and pro-inflammatory cytokines [33,37,38,39,40,41]. It reduces cellular oxidative stress response by scavenging free radicals thus restoring cell viability and protecting cells from apoptosis [29,42,43,44]. In addition, NAC can break down the biofilm of relevant pathogens [33,45,46,47,48]. These antimicrobial/antibiofilm properties make NAC a potential therapeutic drug for oral diseases [33].

Since the oral cavity may contain toxic chemicals, microbial infections, and mechanical cues capable of generating oxidative stress and inducing inflammation [49,50,51], it is essential to maintain the physiological functionality of teeth during an injury and/or infection of the pulp tissue. This accentuates a need for a multifunctional agent capable of neutralizing oxidative stress and modulating inflammatory responses to resume the healing and growth of the injured tissue.

The immune system plays a key role in tissue regeneration. Cellular mechanisms associated with the inflammatory stage are essential for the proper development and function of the pulp tissue. Once these mechanisms are compromised, the functional ability of pulp tissue to reconstruct may be drastically diminished and the pulp is destructed by apoptosis or necrosis.

Excessive inflammation inhibits tissue regeneration and compromises the ability to heal injured tissues. Preserving the vitality of pulp tissue and its physiological function by modulating the inflammatory and stress responses of the cells is, therefore, of significant interest. Our 3D human dental pulp cell model in the extracellular matrix platform (3D/ECM) physiologically and biochemically resembles the tissue environment and facilitates tissue response to drug therapy [52,53].

In this study, NAC is validated as a potential therapy for rescue treatment in pulp cells affected by the toxic concentrations of TEGDMA. Growth recovery and reinstatement of inflammatory and oxidative stress levels are measured and determined by employing our 3D/ECM model. To assess the potential of NAC as a therapeutic drug, its impact on 1) pulp tissue structural regrowth, development and/or preservation of, and 2) the inflammatory and oxidative stress levels in the monomer-affected pulp microtissues are determined.

## 2. Results

### 2.1. NAC Supports the Proliferation and Viability of Human Dental Pulp Cells Affected by TEGDMA

**Figure 1:**
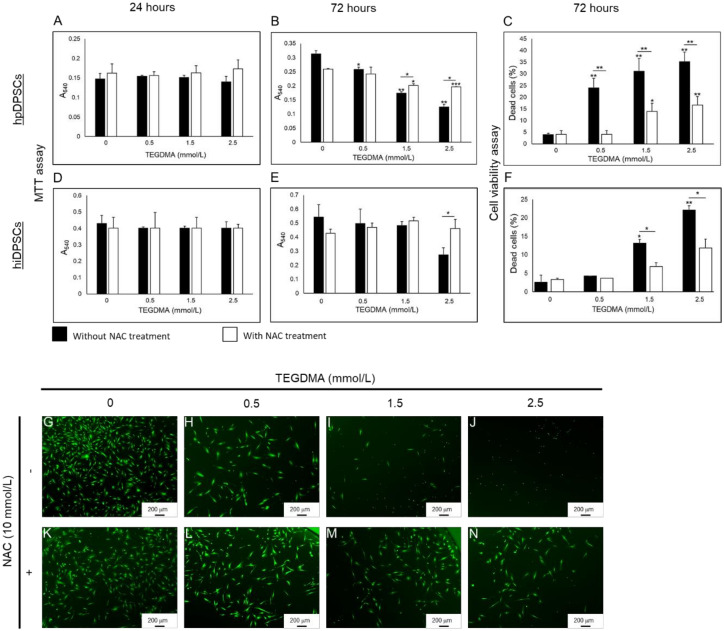
Viability and proliferative capacities of dental pulp cells exposed to TEGDMA and treated with NAC. (**A**–**F**) Bar graphs compare the relative quantities of viable metabolically active (**A**–**C**) hpDPSCs and (**D**–**F**) hiDPSCs. Cell proliferation is determined at 0, 0.5, 1.5, and 2.5 mmol/L TEGDMA after (**A**,**D**) 24 h, and (**B**,**E**) 72 h via MTT analysis. (**C**,**F**) Viability is determined by using live staining and calculating the percentage of dead cells after 72 h of incubation. * *p* ≤ 0.05, ** *p* ≤ 0.01, *** *p* ≤ 0.001. (**G**–**N**) Fluorescence images of NAC (**G**–**J**) non-treated and (**K**–**N**) treated stained hpDPSCs with (**G**,**K**) 0, (**H**,**L**) 0.5, (**I**,**M**) 1.5, and (**J**,**N**) 2.5 mmol/L.

After a 24-h incubation with triethyleneglycol dimethacrylate (TEGDMA), the proliferation of human primary dental pulp stem cells (hpDPSCs) was unaffected compared to cells not exposed to the monomer (Figure 1A). However, after a 72-h incubation at low, moderate, and high concentrations of TEGDMA (0.5, 1.5, and 2.5 mmol/L, respectively) a significant decrease of (0.06, 0.14, and 0.19 absorbance units (AU), respectively; (0.01 ≤ *p* ≤ 0.05)) was observed. A significant decrease of 0.01 and 0.06 AU (*p* ≤ 0.05 and *p* ≤ 0.001, respectively) was detected in N-Acetyl Cysteine (NAC)-treated samples exposed to moderate and high concentrations of TEGDMA compared to TEGDMA non-exposed, NAC-treated samples. The proliferation rate of cells exposed to moderate and high concentrations of TEGDMA, however, increased significantly (*p* ≤ 0.05; increase of 0.03 and 0.07 AU, respectively) compared to the no-NAC group (Figure 1B). Similar results were obtained with human immortalized dental pulp stem cells (hiDPSCs) showing no significant differences in AU values after a 24-h incubation (Figure 1D). After a 72-h incubation, a significant (*p* ≤ 0.05) decrease of 0.27 AU was detected in cells exposed to a high concentration of TEGDMA compared to the non-exposed ones. However, a significant (*p* ≤ 0.05) increase of 0.19 AU was seen between NAC-treated versus non-treated cells exposed to a high concentration of TEGDMA (Figure 1E). These results indicate that NAC only marginally inhibited the proliferation of both cell types at an incubation period of 72 h.

After a 72-h incubation, the percentage of dead hpDPSCs exposed to low, moderate, and high concentrations of TEGDMA while treated with NAC decreased 5.9-, 2.2-, and 2.1-fold, respectively, (*p* ≤ 0.01) in comparison to the non-treated cells. The increase in the percentage of dead cells for both NAC-treated and non-treated cells correlated with the increase in TEGDMA concentration. Exposure of non-treated cells to low, moderate, and high concentrations of TEGDMA resulted in a 6.1-, 7.9-, and 8.9-fold, respectively, (*p* ≤ 0.01) increase in the percentage of dead cells compared to the non-exposed cells. However, when cells were treated with NAC, only the exposure to moderate and high levels of TEGDMA caused a significant 3.4- and 4.07-fold increase, respectively (*p* ≤ 0.05 and *p* ≤ 0.01), in cell mortality rate compared to non-exposed cells (Figure 1C). Similar results were observed with hiDPSCs. No difference in the percentage of dead cells existed between NAC-treated and non-treated groups exposed to a low concentration of TEGDMA. However, a significant (*p* ≤ 0.05) two-fold decrease in the percentage of dead cells was observed in NAC-treated samples exposed to moderate and high concentrations of TEGDMA, compared to the non-treated group. Increasing TEGDMA concentration to moderate and high correlated well with the 5.2- and 8.8-fold increase in the percentage of mortality, respectively, (*p* ≤ 0.05 and *p* ≤ 0.01) compared to the no-TEGDMA group. However, the observed 2.0- and 3.5-fold increase in the percentage of dead NAC-treated cells exposed to the same TEGDMA levels was not statistically significant ((*p* > 0.05); Figure 1F). Fluorescence images of hpDPSCs exposed to the increasing concentrations of TEGDMA, revealed a gradual decrease in the number of viable cells and a change in their morphology when treated with low, moderate, and high concentrations of TEGDMA (Figure 1G–J). However, upon NAC introduction, the viability of the cells increased, and their structures resembled the morphology of the control cells not exposed to TEGDMA (Figure 1K–N).

### 2.2. NAC Resumes the Development of Growth-Arrested Microtissues Exposed and Pre-Exposed to TEGDMA

**Figure 2:**
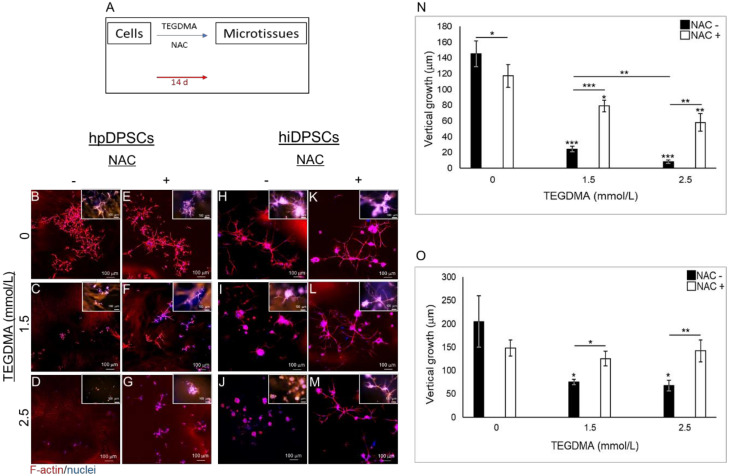
The spatial structure of developing microtissues exposed to TEGDMA and treated simultaneously with NAC. (**A**) Schematic diagram of the experimental assay. (**B**–**M**) Confocal images of (**B**–**G**) hpDPSCs and (**H**–**M**) hiDPSC microtissues (**B**,**E**,**H**,**K**) non-exposed versus exposed to (**C**,**F**,**I**,**L**) moderate and (**D**,**G**,**J**,**M**) high levels of TEGDMA treated with (**E**–**G**,**K**,**L**,**M**) NAC. (**N**–**O**) Bar graphs compare the thicknesses of (**N**) hpDPSCs and (**O**) hiDPSCs structures non-treated vs. treated with NAC. * *p* ≤ 0.05, ** *p* ≤ 0.01, *** *p* ≤ 0.001.

hpDPSCs and hiDPSCs were exposed to the monomer and treated with NAC simultaneously to determine the impact of the drug on the growth and development of cells, injured by TEGDMA, into 3D microtissue structures as described schematically in Figure 2A. Treatment with NAC did not affect hpDPSCs spatial development when compared to the non-treated ones (Figure 2B vs. Figure 2E). Cells exposed to moderate concentration of the monomer were growth-arrested and created separate cell aggregates (spheroids). The introduction of NAC enabled the spheroids to continue their development and interact to establish intact cell layers that represent advanced stages of microtissue formation (Figure 2C vs. Figure 2F). Increasing the concentration of TEGDMA to a higher level interfered with the aggregation of individual cells and led to the production of small undeveloped spheroids. The addition of NAC triggered these spheroids to grow and start interacting into structures representing the early stages of microtissue formation (Figure 2D vs. Figure 2G). The significant decrease in the thicknesses of layers exposed to moderate and high TEGDMA concentrations (3.2- and 7.0-fold; *p* ≤ 0.01) correlated with the increase of the monomer concentration going from the non-treated to NAC treated group. A significant (*p* ≤ 0.01) 6- and 17.5-fold decrease was observed in the thicknesses of cell layers exposed to moderate and high concentrations of the monomer compared to the non-exposed ones, respectively. The thicknesses of NAC-treated layers exposed to moderate and high concentrations of the monomer vs. the ones not exposed to TEGDMA decreased 1.4- and 2.0-fold (*p* ≤ 0.05 and *p* ≤ 0.01), respectively (Figure 2N). In the absence of TEGDMA, the thicknesses of NAC-treated microtissues compared to the non-treated ones showed a significant decrease (*p* ≤ 0.05) of 27 µm. Similar results were obtained for the hiDPSCs structures (Figure 2H vs. Figure 2K; Figure 2I vs. Figure 2L; Figure 2J vs. Figure 2M). A significant (*p* ≤ 0.05 and *p* ≤ 0.01) 1.6- and 2.0-fold decrease was detected in the thicknesses of cell layers exposed to moderate and high concentrations of the monomer, not treated versus NAC-treated, respectively. The thicknesses significantly decreased (2.7- and 3.0-fold, respectively; *p* ≤ 0.05) in cell layers exposed to moderate and high levels of the monomer, compared to the non-exposed layers. The observed decrease in thickness correlated with the increasing TEGDMA concentrations. No significant differences were observed between the thicknesses of NAC-treated layers exposed to moderate and high concentrations of the monomers and the non-exposed ones (Figure 2O).

**Figure 3:**
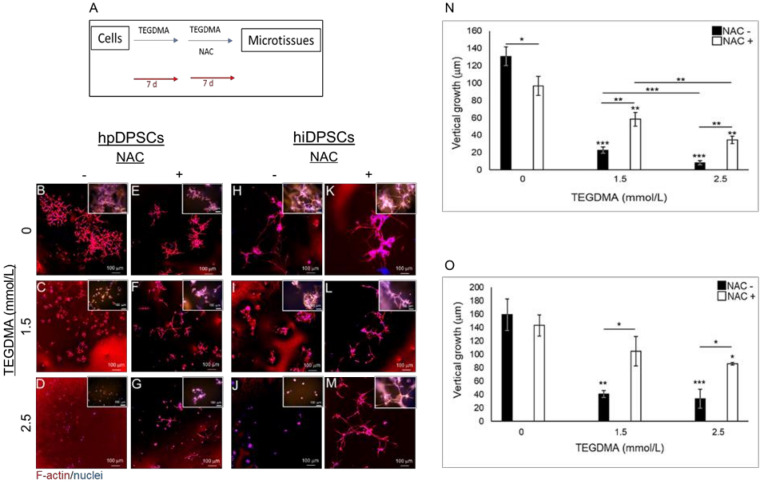
The spatial structure of developing microtissues pre-exposed to TEGDMA prior to treatment with NAC. (**A**) Schematic diagram of the experimental assay. (**B**–**M**) Confocal images of (**B**–**G**) hpDPSC and (**H**–**M**) hiDPSC microtissues (**B**,**E**,**H**,**K**) non-exposed versus exposed to (**C**,**F**,**I**,**L**) moderate and (**D**,**G**,**J**,**M**) high concentrations of TEGDMA before (**E**–**G**,**K**,**L**,**M**) treatment with NAC. (**N**–**O**) Bar graphs comparing the thickness of the (**N**) hpDPSCs and (**O**) hiDPSCs structures non-treated vs. treated with NAC. * *p* ≤ 0.05, ** *p* ≤ 0.01, *** *p* ≤ 0.001.

**Figure A1:**
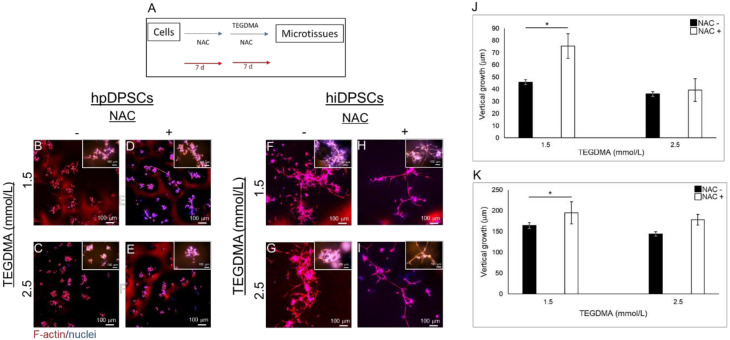
The spatial morphology of developing microtissues treated with NAC prior exposure to TEGDMA. (**A**) Schematic diagram of the experimental assay. (**B**–**I**) Confocal images of (**B**–**E**) hpDPSC and (**F**–**I**) hiDPSC microtissues (**B**,**C**,**F**,**G**) non-treated and (**D**,**E**,**H**,**I**) treated with NAC before exposed to (**B**,**D**,**F**,**H**) moderate and (**C**,**E**,**G**,**I**) high concentrations of TEGDMA. (**J**–**K**) Bar graphs compare the thicknesses of (**J**) hpDPSCs and (**K**) hiDPSCs non-treated vs. treated with NAC. * *p* ≤ 0.05.

The impact of NAC as a rescue therapeutic agent was further validated on the developing microtissues pre-exposed to TEGDMA prior to the drug administration as described schematically in Figure 3A. Pre-exposure of the cells to TEGDMA before NAC-treatment did not prevent the recovery of the cells and the results were similar to the ones obtained from simultaneous incubation of the cells with TEGDMA and NAC. NAC-treated hpDPSC cell layers showed smaller and discontinued branched structures versus the continuous architecture of the non-treated microtissues (Figure 3B vs. Figure 3E). hpDPSCs exposed to moderate concentration of TEGDMA developed into individual separated spheroids. In contrast, cells treated with NAC developed into structures representing advanced stages of microtissue formation where spheroids interacted with the interconnecting cells (Figure 3C vs. Figure 3F). Increasing the concentration of TEGDMA to high levels without treatment with NAC prevented cell–cell interactions. These cells continued to develop and create layers representing the initial stages of microtissue formation and intercellular interactions upon treatment with the drug (Figure 3D vs. Figure 3G). A similar effect could be observed in NAC-treated versus non-treated hiDPSCs samples. In contrast to hpDPSCs, no effect was noticed on the morphology of microtissues treated solely with the drug (Figure 3H vs. Figure 3K). A morphological alternation was demonstrated in the structures of individually separated spheroids exposed to a moderate concentration of TEGDMA which, upon treatment with the drug, started to interact and spread in the matrix creating structures representative of the advanced stages of microtissue formation (Figure 3I vs. Figure 3L). Increasing the concentration of TEGDMA to a higher level revealed again individual and small undeveloped spheroids which, upon treatment with the drug, continued to grow, develop, and interact into structures typical of the advanced stages of microtissue formation (Figure 3J vs. Figure 3M). Quantitative data analysis demonstrated a significant decrease (34 µm; *p* ≤ 0.05) in the thicknesses of NAC-treated versus non-treated microtissues not exposed to TEGDMA. A gradual and significant (5.7- and 15.6-fold, respectively; *p* ≤ 0.001 and *p* ≤ 0.01) decrease was observed in the thicknesses of cell layers exposed to moderate and high concentrations of the monomer compared to the non-exposed ones. A significant 2.7-fold (p ≤ 0.001) decrease in the thicknesses of layers exposed to high versus moderate concentrations of the monomer was also detected. The thicknesses of NAC-treated layers exposed to moderate and high concentrations of TEGDMA, versus the ones not-exposed to the monomer, significantly decreased 1.6- and 2.8-fold, respectively, (*p* ≤ 0.001). A significant 1.7-fold decrease (*p* ≤ 0.01) was also observed between NAC-treated microtissues exposed to moderate versus high concentrations of the monomer. A significant 2.5- and 4.1-fold decrease (*p* ≤ 0.01) was observed in the thicknesses of layers exposed to moderate and high concentrations of TEGDMA in NAC non-treated versus NAC-treated cells (Figure 3N). Similar results were observed for hiDPSCs microtissues. Non-significant differences were observed in the thicknesses of NAC-treated versus non-treated microtissues. A significant 4- and 4.7-fold (*p* ≤ 0.01 and *p* ≤ 0.001) decrease was observed in the thicknesses of cell layers exposed to moderate and high concentrations of the monomer, compared to the non-exposed microtissues. A gradual 1.3- and 1.6-fold (*p* ≤ 0.05) decrease was seen in the thicknesses of NAC-treated layers exposed to moderate and high concentrations of the monomer, respectively, versus the non-exposed microtissues. A significant 2.5-fold (*p* ≤ 0.05) decrease was detected in the thicknesses of layers exposed to moderate and high concentrations of the monomer non-treated versus NAC-treated ones, respectively (Figure 3O). The role of NAC in preventing the cytotoxic effect of TEGDMA on developing microtissues was determined by introducing NAC to the cells before their exposure to TEGDMA (Figure A1A, Appendix A). hpDPSC spheroids exposed to moderate concentration of the monomer showed a slight effect of NAC by starting to interact and to create spreading microtissues (Figure A1B vs. Figure A1D, respectively). However, NAC had no effect on cells exposed to a high concentration of TEGDMA. These cells retained their aggregated form without the ability to interact (Figure A1C vs. Figure A1E, respectively). hiDPSC microtissues exposed to moderate and high concentrations of TEGDMA were slightly affected by the NAC treatment. They transformed from a compact conformation into spreading microtissues within the matrix (Figure A1F vs. Figure A1H,G vs. Figure A1I). Quantification of the data showed a significant increase in the thickness of hpDPSC and hiDPSC layers (29.62 and 27.9 µm, respectively; *p* ≤ 0.05) exposed to a moderate concentration of the monomer after pre-treatment with the drug compared to the non-treated ones (Figure A1J,K).

### 2.3. NAC Prevents Structural Deterioration of Developed and Matured Microtissues Exposed to TEGDMA

**Figure 4:**
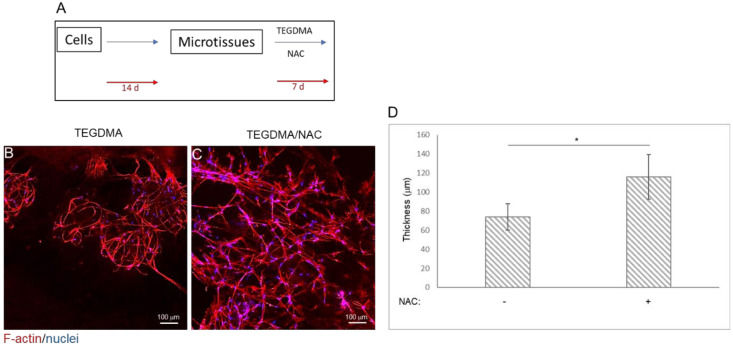
The spatial morphology of injured mature pulp microtissues rescued from structural deterioration by NAC treatment. (**A**) Schematic diagram of the experimental assay. (**B**–**C**) Confocal images of mature microtissues exposure to 2.5 mmol/L TEGDMA and (**B**) non-treated versus (**C**) treated with NAC. (**D**) Bar graph expresses the thickness of the non-treated vs. treated microtissues. * *p* ≤ 0.05.

The impact of NAC on the spatial structure and conformation of mature and developed hpDPSCs microtissues exposed to a high concentration of TEGDMA is presented in Figure 4A. TEGDMA altered the 3D conformation of the microtissue by deteriorating and disassembling the cell layers into smaller disconnected spherical-like layers (Figure 4B). In contrast, NAC-treated microtissues maintained their intact and branched conformation (Figure 4C). Microtissues exposed to the monomer and not treated with NAC showed a significant (*p* ≤ 0.05) decrease in their thickness (73.83 ± 13.84 µm) compared to the NAC-treated ones exposed to the monomer (117.31 ± 22.35 µm) (Figure 4D).

### 2.4. Modulation of the Inflammatory Response Correlates to the Regrowth and Development of TEGDMA-Affected Microtissues Treated by NAC

**Figure 5:**
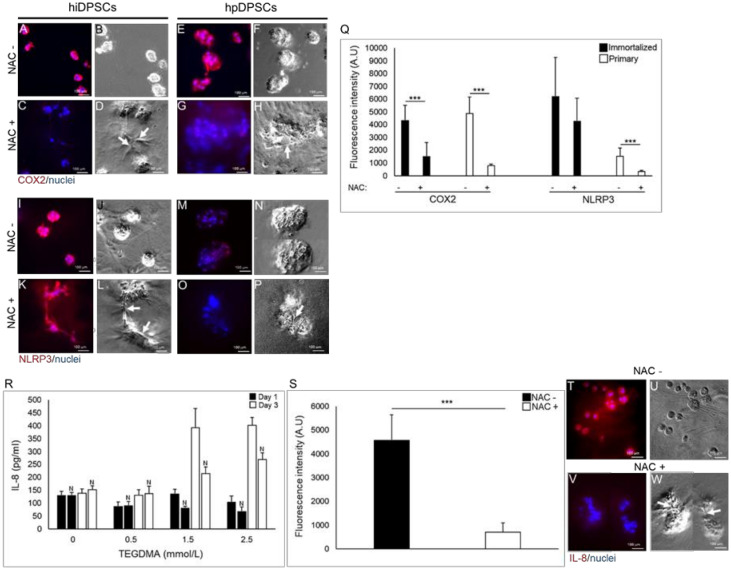
The expression of inflammatory factors in arrested versus rescued pulp microtissues. (**A**,**C**,**E**,**G**,**I**,**K**,**M**,**O**) Fluorescence stock photos and (**B**,**D**,**F**,**H**,**J**,**L**,**N**,**P**) phase-contrast images of (**A**,**B**,**E**,**F**,**I**,**J**,**M**,**N**) growth-arrested spheroids versus (**C**,**D**,**G**,**H**,**K**,**L**,**O**,**P**) rescued (growth-resumed) pulp microtissues treated with NAC of (**A**–**D**,**I**–**L**) hiDPSCs and (**E**–**H**,**M**–**P**) hpDPSCs expressing (**A**–**H**) COX2 and (**I**–**P**) NLRP3. Black arrows point to the interactions between the spherical structures. (**Q**) Bar graph shows the fluorescence intensity of COX2 and NLRP3 in both cell types. *** *p* ≤ 0.001. (**R**) Bar graph expresses the amount of IL-8 released to the medium from the growth-arrested versus rescued microtissues after 1 d and 3 d. “N” denotes NAC treatment and (**S**) the expression level of IL-8 in the cells after a 14-d incubation. *** *p* ≤ 0.001. (**T**,**V**) Fluorescence stock photos and (**U**,**W**) phase-contrast images of IL-8 expression of samples exposed to a moderate concentration of TEGDMA and (**T**,**U**) non-treated versus (**V**,**W**) treated with NAC.

Cyclooxygenase 2 (COX2) was expressed in hiDPSCs (Figure 5A,B) and hpDPSCs (Figure 5E,F) isolated spheroids, exposed to moderate concentration of TEGDMA. The expression could barely be detected when NAC was introduced and cells resumed their growth by changing their morphology and interacting (white arrows) to create microtissues (Figure 5C,D,G,H). The expression levels of COX2, measured by the fluorescence intensity, were significantly decreased 2.8- and 6.1-fold (*p* ≤ 0.001), respectively, in cells exposed to TEGDMA and treated with the drug versus the non-treated cells exposed to the monomer (Figure 5Q).

The expression of the inflammasome NLR family pyrin domain containing 3 (NLRP3) mildly decreased in both types of the developing microtissues (Figure 5K,L,O,P) compared to NLRP3 expression in growth-arrested spheroids exposed to TEGDMA but not treated with NAC (Figure 5I,J,M,N; respectively). A 1.5-fold (non-significant) and 4.7-fold (*p* ≤ 0.001) decrease in the expression (intensity) of NLRP3 in NAC-treated hiDPSCs and hpDPSCs versus the expressions in the non-treated cells, respectively, is shown in Figure 5Q. After a 24-h incubation, the secreted concentration of interleukin 8 (IL-8) filtrate, obtained from the culture mediums of cells exposed to moderate and high concentrations of TEGDMA, decreased by 54.79 and 37.39 pg/mL in cells treated with NAC compared to the non-treated ones, respectively. The decrease in the concentration of the secreted cytokine in the NAC-treated samples was more meaningful after three days (d) of incubation and it reached 178.95 and 133.69 pg/mL for moderate and high concentrations of TEGDMA, respectively, when compared to the non-treated samples (Figure 5R). No change in IL-8 concentration was observed between NAC-treated and non-treated cells exposed to a low concentration of TEGDMA. The expression of IL-8 in hpDPSCs exposed to a moderate concentration of TEGDMA and introduced to NAC decreased significantly (6.4-fold; *p* ≤ 0.001) compared to its expression in the exposed cells not treated with the drug (Figure 5S). Immunocytochemical analysis demonstrated the decrease in the expression of IL-8 in the exposed microtissues which resumed their growth and interconnections (white arrows) upon treatment with NAC (Figure 5V,W) compared to the non-treated/individual spheroids exposed to the monomer (Figure 5T,U).

### 2.5. NAC Moderates the Expression of the Anti-Oxidative Response Elements in TEGDMA-Affected Microtissues

**Figure 6:**
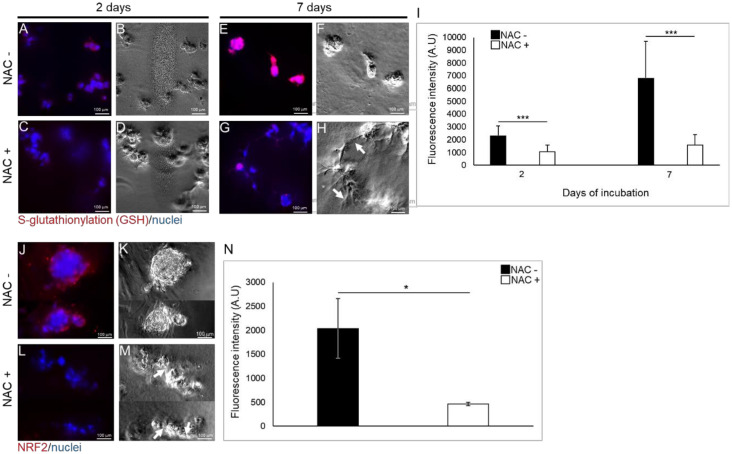
The levels of S-glutathionylation and Nrf2 in growth-arrested versus rescued pulp microtissues at early and advanced stages of development. (**A**,**C**,**E**,**G**) Fluorescence stock photos and (**B**,**D**,**F**,**H**) phase-contrast images of growth-arrested spherical microtissues after (**A**,**B**) 2 d and (**E**,**F**) 7 d versus growth-resumed pulp microtissues treated with NAC after (**C**,**D**) 2 d and (**G**,**H**) 7 d. White arrows point to the interactions between the spherical structures. (**I**) Bar graph measuring the fluorescence intensity of the GSH marker (S-glutathionylated proteins). *** *p* ≤ 0.001. (**J**,**L**) Fluorescence and (**K**,**M**) phase-contrast images of (**J**,**K**) growth-arrested spheroids versus (**L**,**M**) growth-resumed pulp microtissues treated with NAC. White arrows point to the interactions between the spherical structures. (**N**) Bar graph measuring the fluorescence intensity of Nrf2 marker. * *p* ≤ 0.05.

After a two day incubation, expression of reduced glutathione (GSH) conjugated by disulfide linkages to cysteine residues of S-glutathionylated protein in hpDPSCs exposed to moderate concentrations of TEGDMA without the treatment of the drug (Figure 6A,B) was low compared to GSH expression in NAC-treated cells exposed to the monomer (Figure 6C,D). However, after a seven -day incubation, growth-arrested and separated spheroids not treated with NAC showed increased levels of GSH (Figure 6E,F) compared to GSH levels in NAC-treated interacting/developing (pointed by the white arrow) microtissues exposed to the monomer (Figure 6G,H). Fluorescence intensity measurements revealed a significant, two-fold decrease (*p* ≤ 0.001) of the GSH levels in the NAC-treated versus non-treated cells exposed to the monomer after a two-day incubation. These levels continued to decrease significantly after seven days of incubation (4.3-fold; *p* ≤ 0.001). While the levels of GSH increased almost three-fold in the non-treated cells, an increase of less than 1.5-fold was observed in the NAC-treated cells from the second to the seventh day (Figure 6I).

Nuclear factor erythroid-2-related factor 2 (Nrf2) expression was induced in individual growth-arrested spheroids exposed to moderate concentration of TEGDMA without the capability to interact (Figure 6J,K). However, Nrf2 was barely observable in the growth-resumed and interacting (white arrows) microtissues (Figure 6L,M). A significant decrease in Nrf2 levels (4.3-fold; *p* ≤ 0.05) was detected in cells exposed to TEGDMA and treated with NAC versus the non-treated ones exposed to the monomer (Figure 6N).

## 3. Discussion

NAC, a pluripotent drug with antioxidative and immunomodulatory functions [33] has multiple clinical applications in various fields and the list of conditions it can potentially improve has been increasing over the years [36,54]. In this study, the impact of NAC was correlated with the severity of the damage caused by the leachable dental monomer (TEGDMA) by assessing NAC’s ability to restore cell proliferation and reduce the percentage of dead cells when cells are exposed to moderate (1.5 mmol/L) and high (2.5 mmol/L) concentrations of TEGDMA. Previous studies reported the impact of resin composites including the released TEGDMA from pulp capping materials in proximity to pulp tissue. The leachable substance, reaching up to 4 mmol/L, impeded the growth and healing of pulp tissue and induced tissue inflammation [13,14]. However, no meaningful effect was observed in cells exposed to low concentration (0.5 mmol/L) of TEGDMA. NAC resumed the 2D structural development of cells at increasing concentrations of the monomer. In 3D cultures, it resumed the spatial growth, interactions, and assembly of individual cells exposed to high concentrations of TEGDMA into spheroids. NAC continued the growth and development of cells, exposed to moderate concentration of the monomer, to a higher level of organization by supporting adjacent spheroids to interact and create continuous multicellular layers (microtissues). A previous study has demonstrated the capability of NAC to attenuate the reduction of cell viability caused by the photoinitiator used in dental composites—camphorquinone [55].

Our findings accentuate the assumption that NAC supports cell–cell and cell–substratum adhesions probably through the involvement and mobilization of E-cadherin and β-catenin from the nucleus to the adherent junctions [56,57]. The impact of NAC on the structural recovery of hiDPSCs was greater than hpDPSCs due to the aggressive growth of immortalized cells and their capability to recover faster. Similar results were observed in cells pre-exposed to high concentrations of TEGDMA before the treatment with NAC thus demonstrating the capacity of NAC to recover cells that were already under the toxic influence of TEGDMA. Although the affected cells treated with NAC have not reached the complexity and size of the non-exposed controlled microtissues, significant progress has been reached. These observations lead to the assumption that NAC may initiate the healing process of the injured cells. Further studies should be conducted to test the impact of NAC in combination with other regenerative therapies on the recovery process of the pulp tissue. Our data are consistent with other reports showing the ability of NAC to attenuate growth inhibition of cells treated with Antimycin A [58], which possess inhibitory mechanisms similar to TEGDMA, or when exposed to hydrogen peroxide (H_2_O_2_) [59]. One possible explanation suggested that NAC suppresses the elevated ROS levels while the alternative would suggest a restoration of matrix metalloproteinases (MMPs) activity by NAC by preventing MMPs degradation. We found that the NAC impact was expressed less in cells pre-treated with the drug prior to their exposure to TEGDMA. Furthermore, NAC only slightly inhibited the development of the control microtissues not exposed to TEGDMA. This would suggest that NAC should not be considered under normal conditions where the tissue is not at risk. NAC was capable of protecting the mature microtissues from the deteriorating effect of the monomer. This ability of NAC to defend tissues and their components from destruction is relatable to its roles in preventing oxidative degradation of biological membranes and/or degradation of MMPs [60,61].

Resin composite restorations may trigger the inflammatory response in dental pulp tissue by inducing COX-2 expression, leading to the production and release of prostaglandin E2 (PGE_2_) and the pro-inflammatory cytokine interleukin-8 (IL-8) [55,62,63,64]. COX-2 was expressed in both hpDPSCs and hiDPSCs undeveloped spheroids exposed to the monomer. NAC administration decreased the expression of the enzyme in the recovered spheroids that continued to interact and create the microtissues. Our data correlate well with studies demonstrating the downregulatory effect of NAC on COX-2 and PGE_2_ via the inhibition of the inducer interleukin 1β (IL-1β) [63,65]. Production of IL-1β is dependent on the assembly of the NLR family pyrin domain containing 3 (NLRP3) inflammasome and it initiates a cascade that promotes the secretion of other pro-inflammatory products including IL-8 [66,67,68]. In this study, we demonstrated a similar pattern for both cell types. hpDPSCs and hiDPSCs expressed the inflammasome when exposed to TEGDMA and reduced its expression levels upon treatment with NAC. Excessive activation of the inflammasome strongly correlates with the inflammatory diseases. It is mediated by IL-1β [69]. These alternations of the immune response may lead to an inflammatory reaction and hypersensitivity that have previously been reported after the placement of new resin-based composites and adhesives. The induction of NLRP3 and formation of the cytokines IL-1β and IL-18 in human peripheral blood mononuclear cells (hPBMCs) was demonstrated by resin monomers TEGDMA and 2-hydroxyethyl methacrylate (HEMA) [67,70]. Although the expression of IL-18 requires further study, NLRP3/caspase-1 was reported to exhibit a biological role in the innate immune response of human dental pulp fibroblasts leading to activated IL-1β and IL-18 [71,72]. Since the generation of ROS can activate the inflammasome by leading to caspase-1 activation and IL-1β secretion, NAC is speculated to block this effect by inhibiting the activation of NLRP3 [73] and attenuating the expression of both downstream components [74]. Differences in the expression levels of NLRP3 were detected between the two cell types with a significant decrease of NLRP3 in NAC-treated hiDPSCs. The explanation for the observed differences might be related to the differences of the redox state at baseline and the sensitivity to reducing agents exhibited by primary versus the immortalized cells. NLRP3 induction rate is dependent on ROS production [75,76]. The expression of intracellular IL-8 was elevated in TEGDMA-affected spheroids. However, this expression in the developing microtissues was reduced upon NAC administration. Similar trends were observed in the extracellular cytokine levels secreted by the affected cells treated versus cells not treated with the drug. NAC prevented the gradual elevation of IL-8 secretion over time. Significantly, data from previous studies demonstrated the capacity of NAC to prevent the expression of induced pro-inflammatory cytokines including IL-8 in other cell types such as macrophages and gingival fibroblasts [77].

TEGDMA-induced cytotoxicity is reportedly associated with an early and drastic depletion of cellular radical scavenger GSH in pulpal fibroblasts, followed by the production of ROS and disruption of the redox balance. These processes led to the overproduction of ROS, cell cycle arrest, and eventually induced cell death via apoptosis [78,79,80]. NAC was found to moderate the oxidative stress response by partially inhibiting GSH depletion in cells treated with 0.5 mmol/L TEGDMA, protecting the pulp and the surrounding tissues from the toxic effect of dental restoratives [17,81,82,83]. NAC was also shown to reduce ROS formation via the attenuation of the cyclin-dependent kinase inhibitor (p21) and the heme oxygenase (HO-1) stimulated by the resin composite [55]. In our system, TEGDMA increased GSH levels over time in the growth-arrested spheroids. In contrast, the addition of NAC kept GSH at low and stable levels in the growing and developing microtissues. The significant depletion of the intracellular GSH pool by TEGDMA is assumed to be an early process. At prolonged incubation times with the monomer, the recovery of GSH at TEGDMA concentrations ≥ 2.5 mmol/L was observed. It is speculated that the upregulation of GSH synthesis came in response to its initial depletion. Low levels of GSH induced its de-novo synthesis to increase the physiological concentration. Thus, the intracellular levels may fall temporarily but they are rapidly restored under normal conditions. However, if the regulatory increase of GSH is not sufficient to maintain the intracellular levels required for normal metabolism upon exposure to the monomer, the depletion will lead to cell death. The support for the above-proposed mechanism could be found in the fact that TEGDMA influences the metabolic events rather than having a direct toxic impact on the tissue and NAC’s regulatory role may change these events and save cells from death [84]. Since ROS oxidize certain cysteine residues of target proteins, GSH is utilized to reduce these residues by conjugating to the cysteine moieties forming S-glutathionylated proteins via disulfide linkages. This process leads to GSH depletion and enhances the oxidative state of the cell. NAC is known to replenish GSH by disfavoring the S-glutathionylation reaction [85,86] and stimulating the breakage of disulfides thus restoring free thiols known for their antioxidative activity and increased synthesis of GSH [34]. The gene involved in GSH biosynthesis is positively regulated by the nuclear factor (NF) erythroid 2-related factor 2 (Nrf2) via the decrease in the synthesis of GSH synthetase [87]. Induction of Nrf2 is known to play an important role in the protection of cells and tissues from oxidative damage as observed in RAW267.4 macrophages exposed to toxic chemical extracts [88]. The levels of Nrf2 in the non-developed spheroids exposed to TEGDMA were significantly decreased when cells were treated with NAC and continued to develop. Our observations are in agreement with a report demonstrating the downregulatory effect of NAC on the H_2_O_2_-mediated activation of the Nrf2/HO-1 pathway [89]. High levels of Nrf2 in macrophages exposed to the dental resin monomer HEMA were found to correlate with the activation of the factor as a protective response against the chemical substance [90]. A different approach claims that, since Nrf2 is essential for inflammasome activation, NAC may partially modulate the inflammatory response through the inhibition of this factor [91]. A similar inhibitory effect was observed in human keratinocyte ovarian cancer cells exposed to H_2_O_2_ and treated with NAC [92]. Further studies are needed to explore the activation and identity of antioxidant genes targeted by Nrf2 by using chromatin immunoprecipitation and investigate their regulatory role in the growth of compromised microtissues upon treatment with NAC.

The use of NAC modulatory capabilities to alleviate the inflammatory and oxidative state of challenged pulp is part of a growing effort to facilitate the growth, healing, and function of the tissue [93,94].

To the best of our knowledge, this is the first study showing that NAC resumes growth and promotes the interaction of compromised human dental pulp cells to create 3D microtissue in an extracellular environment. Most studies on NAC reported in the literature are performed in 2D cultures lacking the capabilities to follow cell–cell interaction in a biomimetic environment such as the natural extracellular matrix. Our 3D system demonstrated the capabilities of NAC to (i) resume growth and development of cells pre-exposed and damaged by TEGDMA, (ii) protect the mature structure from deterioration by the chemical substance, and (iii) alleviate the inflammatory and oxidative stress responses associated with the developmental recovery of the cells in ECM and creation of microtissues.

## 4. Materials and Methods

### 4.1. Cell Cultures

Human primary dental pulp stem cells (hpDPSCs) were isolated from pulp tissues removed from third molars. The molars were obtained from healthy patients (aged 20 to 40 years) undergoing extraction at the NIH Dental Clinic and informed consent was collected according to the guidelines. The study was reviewed and approved by the American Dental Association (ADA) Institutional Review Board and was not considered to be human subject research. The teeth were broken into pieces in a sterile environment, exposing the pulp tissue in the pulp chamber and root canals. The pulp was gently isolated by using sterile tweezers. The excised pulp tissue was incised into 1 to 2 mm^2^ pieces and incubated in a T-25 flask with DPSC BulletKit^TM^ Medium (Lonza, Walkersville, MD, USA). Medium was replenished every 2 to 3 d. Cells outgrown from the pulp tissue explants [95] were collected after 14 to 21 d. The cells were previously characterized and found to express the self-renewal and mesenchymal stem cell markers: sex determined region y-box 2 (SOX2), octamer-binding transcription factor 4 (OCT4), and CD105, respectively. They were capable of differentiating and depositing the mineral in the calcifying medium [96]. Human immortalized dental pulp stem cells (hiDPSCs; TP-023 [97]) were obtained as a gift from Dr. Lawrence T. Reiter, the University of Tennessee Health Science Center (UTHSC), Memphis, TN, USA and approved by the UTHSC Institutional Review Board. Informed consent was obtained according to the guidelines. Both DPSCs were cultured in Dulbecco’s Modified Eagle Medium (DMEM; Invitrogen, Carlsbad, CA, USA). Media were supplemented with 10% fetal bovine serum, 2 mmol/L L-glutamine, and 100 units/mL of penicillin and 100 µg/mL of streptomycin (Invitrogen, Carlsbad, CA, USA). At approx. 80% confluence, cell cultures were split at 1:5. All incubations were performed in a 5% CO_2_ humidified atmosphere at 37 °C. TEGDMA (Esstech, Essington, PA, USA) was dissolved in ethanol to a stock concentration of 1 mol/L. Before utilization, a fresh 20 mmol/L TEGDMA batch was prepared by diluting the stock solution with the DMEM. TEGDMA solution was then sequentially diluted to the final concentrations of 0.5, 1.5, and 2.5 mmol/L. For 2D cell cultures, 1.7 × 10^4^ cells/well were seeded in a 48-well plate (Greiner bio-one, Monroe, NC, USA).

### 4.2. 3D ECM Cultures

The 3D cell cultures were prepared by coating a 48-well plate or Lab-Tek II Chambered Coverglass (8-well, Nalgene Nunc International, Rochester, NY, USA) surfaces with 100 µL of Matrigel^TM^-extracellular matrix ECM (ECM/BM; Corning, Tewksbury, MA, USA) and incubating for 30 min (min) at 37 °C. 1.7 × 10^4^ cells/well were seeded on the solidified ECM/BM and then incubated for 2 h. The adherent cells were covered with medium containing ECM/BM. After 24 h of incubation, media was replaced with fresh media containing the soluble monomer TEGDMA at different concentrations. Medium without the monomer served as a control. The culture medium was replenished twice weekly. For mature microtissues, TEGDMA was added after the microtissues were developed for 14 d.

### 4.3. Cell Proliferation and Viability

Cell viability was determined by fluorescence microscopy using a LIVE/DEAD cell imaging kit (488/570, Molecular Probes, Eugene, Oregon, USA). Fluorescein and tetramethylrhodamine optical filters were used to identify live and dead cells, respectively. A total of 5 × 10^3^ cells was seeded in a 96-well plate (Greiner bio-one, Monroe, NC, USA) in triplicates and incubated for 24, 48, and 72 h. Next, a 3-(4,5-dimethylthiazol-2-yl)-2,5-diphenyltetrazolium bromide (MTT) assay was performed using the Vybrant MTT cell proliferation assay kit (Invitrogen, Carlsbad, CA, USA) according to the manufacturer’s instructions. Absorbance was measured at 540 nm after a 10 min incubation with the reagent.

### 4.4. Cell Staining

The 2D and 3D hpDPSCs and hiDPSCs cultures were washed twice with Dulbecco’s phosphate-buffered saline (DPBS; Invitrogen) and fixed with 4% paraformaldehyde (Electron Microscopy Sciences, Hatfield, PA). Cells nuclei and F-actin were stained with 0.1 µmol/L phalloidin and 1 µg/mL Hoechst for 30 min (Molecular Probes, Eugene, OR), respectively, after permeabilization with 0.1% Triton X-100 (Alfa Aesar, Ward Hill, MA, USA) for 5 min.

For immunostaining, cells were washed twice with 0.1% bovine serum albumin (Invitrogen), blocked for 45 min with 10% normal donkey serum (GeneTex, Irvine, CA, USA), 0.3% Triton X-100, and 1% bovine serum albumin and then incubated with 10 µg/mL of anti-COX2 (MAB4198), NLRP3 (MAB7578), IL-8 (MAB208), Nrf2 (MAB3925; R&D Systems, Minneapolis, MN, USA), and 50 µg/mL anti-GSH (D8/sc-52399; Santa Cruz, Dallas, TX, USA) monoclonal antibodies overnight at 4 °C. The anti-GSH clone D8 antibody is capable of identifying S-glutathionylated protein moieties. Following the incubation, cells were washed twice and incubated with a 1:200 dilution of donkey anti-mouse Immunoglobulin G (IgG; NL007, R&D Systems) or goat anti-rat immunoglobulin G (NL013, R&D Systems) NorthernLights^TM^ NL557-conjugated polyclonal antibodies, respectively, for an hour at RT. After washing and incubation with 14.3 mmol/L 4′,6-diamidino-2-phenylindole (DAPI; Molecular Probes) for 5 min at room temperature (RT), the cells were washed again and kept in DPBS at 4 °C before being analyzed.

### 4.5. Microscopy and Image Analysis

Fluorescence images of the 2D cultures were taken by a Zeiss Axiovert A1 inverted fluorescence microscope (Carl Zeiss, Jena, Germany) equipped with an AxioCam MRm CCD camera and a, LED excitation light source (Thorlabs, Newton, NJ, USA). Individual cells were counted by using the Zen 2 counting mode (Carl Zeiss). Fluorescence images and intensities of the 3D/ECM hiDPSCs and hpDPSCs immunostained cultures were taken and quantified by stitching sliced images along the z-axis (motorized inverted Eclipse Ti-E epifluorescence microscope; Nikon Instruments Inc., Melville, NY, USA) by using the NIS-Elements software version 3.0 (Nikon Instruments Inc). Cross-sectional analysis of the developing structures was performed by Z-stacking images in each channel into a single image representing both fluorescence channels. The images were collected by laser-scanning confocal microscopy (LCSM; 3.5 µm intervals, two fluorescence channels; Leica Microsystems Inc., Buffalo Grove, IL, USA). To measure the thickness and diameter, 25 to 30 constructs/microtissues were observed randomly per each experimental group. Thicknesses of constructs were measured along both x–z and y–z planes using Leica Application Suite Advanced Fluorescence (LAS AF) software (Leica Microsystems Inc). The 3D structures were created from stacking images obtained by LCSM, and analyzed by Image J software (V.1.48, NIH).

### 4.6. 3D Cell Culture ELISA

Measurements of extracellular IL-8 was obtained from the 3D cultures media and performed with the Human IL-8/CXCL8 DuoSet ELISA (enzyme-linked immunosorbent assay) kit (DY208; R&D Systems) according to the manufacturer’s recommendations.

### 4.7. Statistical Analysis

Viability values and percentage of dead cells in 2D cultures, and 3D structure thicknesses and fluorescence intensities were expressed as mean value ± one standard deviation of at least three separate experiments performed in triplicate. Statistical comparisons were performed using a one-way analysis of variance (ANOVA) followed by a two-tailed Student’s *t*-test for the unpaired samples or followed by Tukey’s post-test for multiple comparisons. Results were considered statistically significant when *p* ≤ 0.05.

## 5. Conclusions

**Figure 7:**
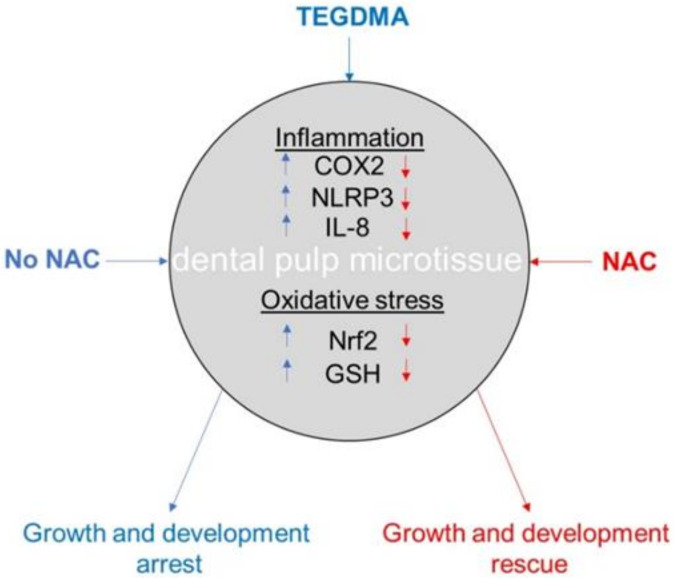
Schematic diagram recapitulates the impact of TEGDMA exposure and NAC treatment on inflammatory and oxidative stress responses of growth-arrested versus resumed (rescued) dental pulp microtissue affected by toxic dental resin monomer. Arrow pointing up and down indicates an increase and decrease in protein levels, respectively.

NAC rescues cells exposed or pre-exposed to moderate and high concentrations of TEGDMA, overcomes their growth-arrest, and protects mature microtissues from structural deterioration. The growth recovery is associated with the modulation and reinstatement of the inflammatory and anti-oxidative responses (Figure 7). The 3D dental pulp cell-based ECM platform employed in this study is a useful tool for screening rescue therapy treatments of injured dental pulp tissues. Our findings may encourage the therapeutic utility of NAC and suggest its integration into restorative dental materials to prevent destructions of compromised pulp tissues and maintain their vitality.

## Abbreviations

ADA	American Dental Association
ANOVA	Analysis of Variance
AU	Absorbance Units
COX2	Cyclooxygenase 2
d	Days
DAPI	6-diamidino-2-phenylindole
DMEM	Dulbecco’s Modified Eagle Medium
DPBS	Dulbecco’s Phosphate-Buffered Saline
ECM	Extracellular Matrix
ELISA	Enzyme-Linked Immunosorbent Assay
FDA	Food and Drug Administration
GSH	Glutathione
H	Hours
HEMA	2-hydroxyethyl methacrylate
hiDPSC	Human Immortalized Dental Pulp Stem Cell
hpDPSC	Human Primary Dental Pulp Stem Cell
H_2_O_2_	Hydrogen Peroxide
IgG	Immunoglobulin G
IL-1β	Interleukin 1β
IL-8	Interleukin 8
LCSM	Laser-Scanning Confocal Microscopy
min	Minutes
MTT	(3-(4,5-dimethylthiazol-2-yl)-2,5-diphenyltetrazolium bromide
NAC	N-Acetyl Cysteine
NLRP3	NLR Family Pyrin Domain Containing 3
Nrf2	Nuclear Factor Erythroid-2-Related Factor 2
OCT4	Octamer-Binding Transcription Factor 4
ROS	Reactive Oxygen Species
SOX2	Sex Determined Region y-Box 2
TEGDMA	Triethyleneglycol Dimethacrylate
UTHSC	University of Tennessee Health Science Center
